# Supplementary material for: Can Near-Infrared Spectroscopy Replace a Panel of Tasters in Sensory Analysis of Dry-Cured Bísaro Loin?
Source: Foods. 2023 Dec 1;12(23):4335. doi: 10.3390/foods12234335 (PMC10706236; doi:10.3390/foods12234335)
Supplement: Supplementary file 1 [file foods-12-04335-s001.zip › foods-2725265-supplementary.pdf]

Table S1. Parameters and figures of merit of the best PLS models obtained for each sensory attribute.

		Calibration *		Prediction *		Durbin-Watson	
**Attribute**	**LVs**	**RMSE**	**R^2**	**RMSE**	**R^2**	**DW**	**pDW**
Odor	8	0.4054	0.3822	0.4705	0.2585	2.260	0.2974
Andros	7	0.2785	0.4959	0.2980	0.4405	1.931	0.6728
Scatol	3	0.3237	0.0847	0.2772	0.1415	1.977	0.8259
Lean color	7	0.3974	0.2614	0.3685	0.1882	1.968	0.7954
Fat color	7	0.4703	0.3509	0.5725	0.0691	2.249	0.3218
Hardness	7	0.5356	0.3822	0.4805	0.2992	2.114	0.6959
Juiciness	9	0.4591	0.6205	0.5378	0.3304	2.101	0.7417
Chewiness	8	0.3726	0.5814	0.3010	0.6644	1.725	0.1745
Flavor intensity	5	0.3211	0.4124	0.3299	0.3738	1.729	0.1851
Flavor persistence	8	0.3428	0.5145	0.3618	0.3811	2.044	0.9384

* All models used MSC pretreatment and autoscaling normalization; MSC- multiplicative scatter correction; Andros- odor androsterone; RMSE- root mean square error; R^2- coefficient of determination; DW- Durbin-Watson value; pDW – p-value for Durbin-Watson test; LVs – latent variables.
